# Surgical Methods and Experiences of Surgeons did not Significantly Affect the Recovery in Phonation Following Reconstruction of the Recurrent Laryngeal Nerve

**DOI:** 10.1007/s00268-016-3634-7

**Published:** 2016-07-18

**Authors:** Kana Yoshioka, Akira Miyauchi, Mitsuhiro Fukushima, Kaoru Kobayashi, Minoru Kihara, Akihiro Miya

**Affiliations:** 1 Department of Head and Neck Surgery, Center for Excellence in Thyroid Care, Kuma Hospital, 8-2-35 Shimoyamate-dori, Chuo-ku, Kobe, 650-0011 Japan; 2 Department of Surgery, Center for Excellence in Thyroid Care, Kuma Hospital, 8-2-35 Shimoyamate-dori, Chuo-ku, Kobe, 650-0011 Japan

## Abstract

**Background:**

We reported phonatory recovery in the majority of 88 patients after recurrent laryngeal nerve (RLN) reconstruction. Here we analyzed factors that might influence the recovery, in a larger patient series.

**Methods:**

At Kuma Hospital, 449 patients (354 females and 95 males) underwent RLN reconstruction with direct anastomosis, ansa cervicalis-to-RLN anastomosis, free nerve grafting, or vagus-to-RLN anastomosis; 47.4 % had vocal cord paralysis (VCP) preoperatively. Maximum phonation time (MPT) and mean airflow rate during phonation (MFR) were measured 1 year post surgery. Forty patients whose unilateral RLNs were resected and not reconstructed and 1257 normal subjects served as controls.

**Results:**

Compared to the VCP patients, the RLN reconstruction patients had significantly longer MPTs 1 year after surgery, nearing the normal values. The MFR results were similar but less clear. Detailed analyses of 228 female patients with reconstruction for whom data were available revealed that none of the following factors significantly affected phonatory recovery: age, preoperative VCP, method of reconstruction, site of distal anastomosis, use of magnifier, thickness of suture thread, and experience of surgeon. Of these 228 patients, 24 (10.5 %) had MPTs <9 s 1 year after surgery, indicating insufficient recovery in phonation. This insufficiency was also not associated with the factors mentioned above.

**Conclusions:**

Approximately 90 % of patients who needed resection of the RLN achieved phonatory recovery following RLN reconstruction. The recovery was not associated with gender, age, preoperative VCP, surgical method of reconstruction, or experience of the surgeon. Performing reconstruction during thyroid surgery is essential whenever the RLN is resected.

## Introduction

Vocal cord paralysis (VCP) and hypoparathyroidism are the most common complications in thyroid surgery, and they are known to compromise the patients’ quality of life. Thyroid cancer often invades the recurrent laryngeal nerve (RLN), causing VCP. Most patients with this condition require resection of the invaded portion of the RLN. Even among thyroid cancer patients whose vocal cords are functional preoperatively, the cancer may involve their RLNs. In such cases we have tried to preserve the nerve with sharp dissection, which sometimes resulted in significant thinning of the preserved nerve that we call ‘a partial layer resection’ of the RLN. Up to 83 % of such patients recovered their vocal cord function [1]. However, a considerable number of such patients have needed segmental resections of the RLN during the surgery. Accidental transection of the RLN may also occur during thyroid surgery, even in simple cases [2].

Transection or segmental resection of the unilateral RLN causes VCP. The symptoms of VCP are hoarseness, shortening of phonation time, and aspiration [3]. Aspiration can be life-threatening, as it can induce aspiration pneumonia, especially in elderly patients [3]. We have reconstructed resected RLNs by various methods, and we reported the recovery of phonatory function in patients with nerve reconstruction, although the vocal cords on the reconstructed side remained immobile [4–7]. This is explained as follows. Although the normal movements of the vocal cords were not restored because of misdirected reinnervation among the adductor fibers and the abductor fibers, the patients’ voices recovered since the reinnervated cords recovered from atrophy, and tension was restored during phonation [8]. Aspiration was also reduced following RLN reconstruction.

We reported that the recovery of phonatory function occurred in 87 % of 88 patients irrespective of age, gender, the presence or absence of preoperative VCP, the method of reconstruction, and the use of surgical magnifiers [7]. However, the number of patients was relatively small for a detailed analysis of factors that might influence the outcome of RLN reconstruction, and the 88 patients were operated on by a single surgeon (A. Miyauchi). In the present report, we analyzed all of the cases of RLN reconstruction performed by 21 surgeons at Kuma Hospital over the past 17 years.

## Patients and methods

### Patients

During the 17-year period from January 1998 to November 2014, 24,757 thyroid surgeries were done at Kuma Hospital. The numbers of primary and redo operations were 23,606 and 1151, respectively. Among these surgeries, 507 patients (2.04 %) underwent resection of a unilateral RLN, with surgery performed for thyroid cancer in 488 patients and for benign thyroid diseases in 19 patients (Table 1). Preoperative laryngoscopic examinations revealed VCP in 231 patients (45.6 %).

**Table 1:** Clinical features of patients who underwent reconstruction of the RLN and patients who underwent resection of the RLN without reconstruction

Total (*n* = 507)	RLN reconstructed (*n* = 449) *n* (%)	RLN resected (*n* = 58) *n* (%)
Age (yrs)		
Mean [SD]	59.2 (14.6)	55.6 (17.1)
Sex		
Female	354 (78.8)	46 (79.3)
Male	95 (21.2)	12 (20.7)
Thyroid disease		
Benign	19 (4.2)	0 (0)
Malignancy	430 (95.8)	58 (100)
VCP preoperatively		
Yes	213 (47.4)	18 (31.0)
No	236 (52.6)	40 (69.0)
Operation		
Primary	422 (94.0)	54 (93.1)
Redo operation	27 (6.0)	4 (6.9)
Transection of RLN		
Invasion of cancer	409 (91.1)	58 (100.0)
Entrapment by benign tumor	3 (0.7)	0 (0)
Accidental	37 (8.2)^†^	0 (0)

*RLN* recurrent laryngeal nerve, *VCP* vocal cord paralysis

^†^0.14 % of all thyroid surgery cases in the study period

The reasons for RLN resection were invasion by cancer, entrapment by a benign tumor, and accidental transection in 467, 3, and 37 patients, respectively. Accidental transections occurred in 0.14 % of all thyroid surgery cases in the study period (Table 1). Of the 507 patients who had resection or transection of the RLN, 449 patients (88.7 %) underwent reconstruction of the RLN in the same operation. However, 58 patients (11.3 %) did not undergo reconstruction of the RLN because the distal stump of the resected RLN could not be identified (*n* = 19 patients), massive local infiltration by tumor (*n* = 21), anaplastic thyroid cancer (*n* = 16), or unclear reasons (*n* = 2). The patients with RLN reconstruction were 354 females and 95 males aged 9–86 years (mean 59.2 years) (Table 1). Preoperative laryngoscopic examinations revealed VCP in 47.4 % (*n* = 231) of these patients.

### Methods of reconstruction of the RLN

**Fig. 1:**
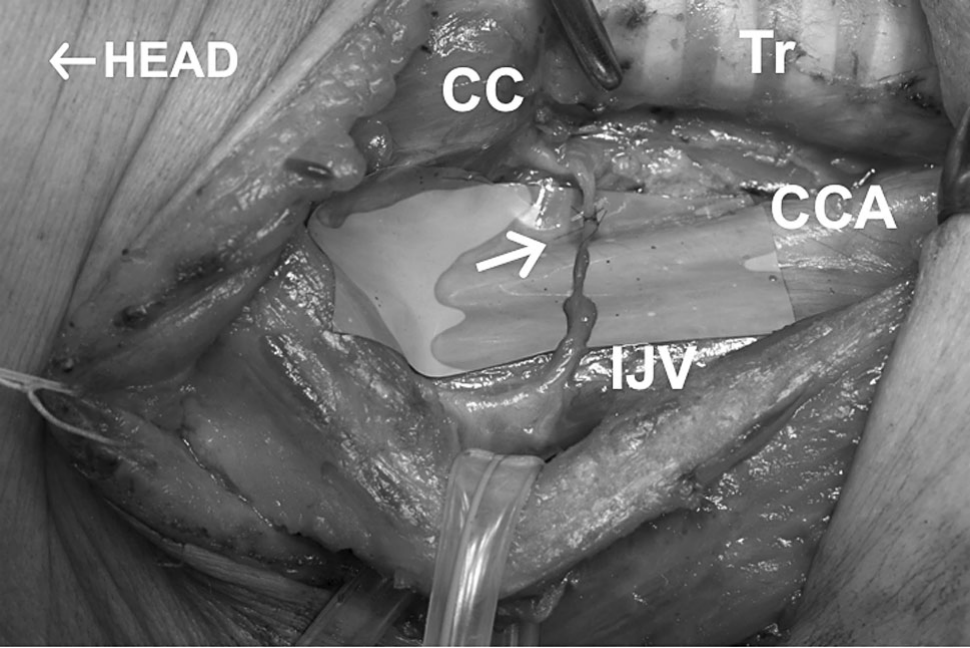
Ansa cervicalis-to-recurrent laryngeal nerve anastomosis. *Arrow* indicates the anastomosis. *CC* the cricoid cartilage, *Tr* trachea, *CCA* common carotid artery, and *IJV* internal jugular vein. Head to the *left*

**Fig. 2:**
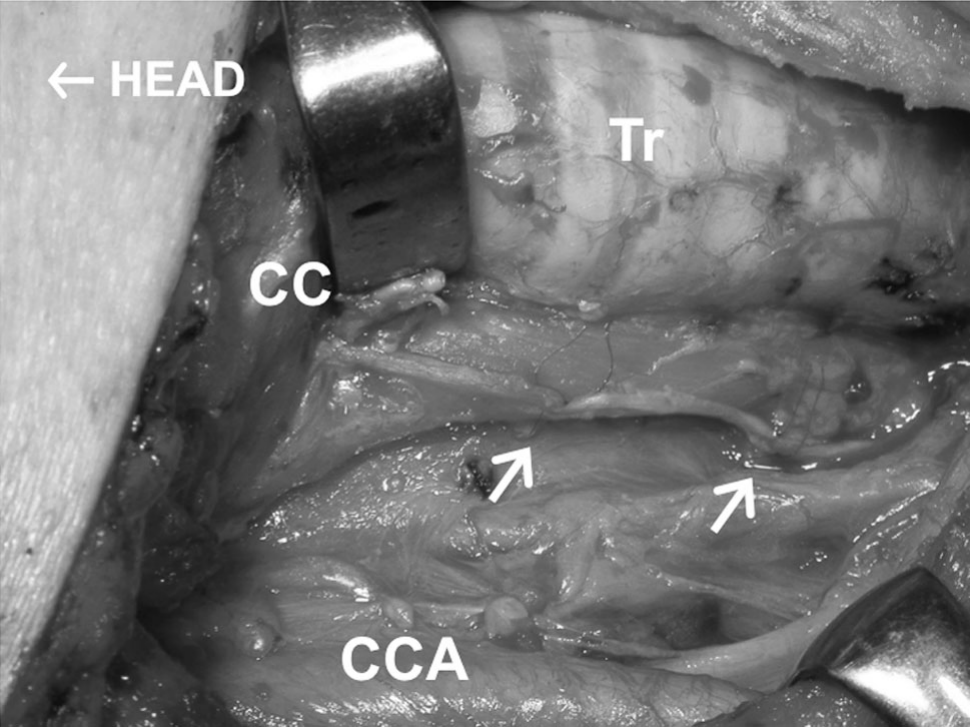
Free nerve grafting. *Arrows* indicate two anastomoses. Head to the *left*

The methods of RLN reconstruction were direct anastomosis (DA) of the cut ends (*n* = 59), ansa cervicalis-to-RLN anastomosis (ARA) (*n* = 345) (Fig. 1), free nerve grafting (FNG) to fill the defect in the RLN (*n* = 35) (Fig. 2), and vagus-to-RLN anastomosis (VRA) (*n* = 10) (Table 2). End-to-end anastomoses were made with one to four stitches with 6–0 to 9–0 monofilament thread using microsurgery instruments with or without surgical loupes × 2.5 magnification. A VRA was performed in patients who had resection of the RLN and the ipsilateral vagus because of cancer invasion.

**Table 2:** Details of the methods of reconstruction of the recurrent laryngeal nerve

	No. of patients *n* = 449 *n* (%)	Site of distal anastomosis	
		Outside^*^ *n* = 349	Inside^**^ *n* = 145
Direct anastomosis (DA)	59 (13.1)	56	3
Ansa cervicalis-RLN anastomosis (ARA)	345 (76.9)	255	90
Free nerve grafting (FNG)	35 (7.8)	30	5
graft taken from:			
ansa cervicalis	23		
supraclavicular nerve	6		
great auricular nerve	6		
Vegas-RLN anastomosis (VRA)	10 (2.2)	8	2

^*^Outside: the distal anastomosis was made outside the thyroid cartilage

^**^Inside: the distal anastomosis was made behind the thyroid cartilage

In the early study period, a single surgeon split the vagus longitudinally and anastomosed its medial portion with the distal stump of the RLN, since the medial portion of the vagus was reported to possibly contain the motor nerve fiber element for the RLN [9]. Currently we do not perform this procedure, because we believe that an ARA is superior to this procedure.

**Fig. 3:**
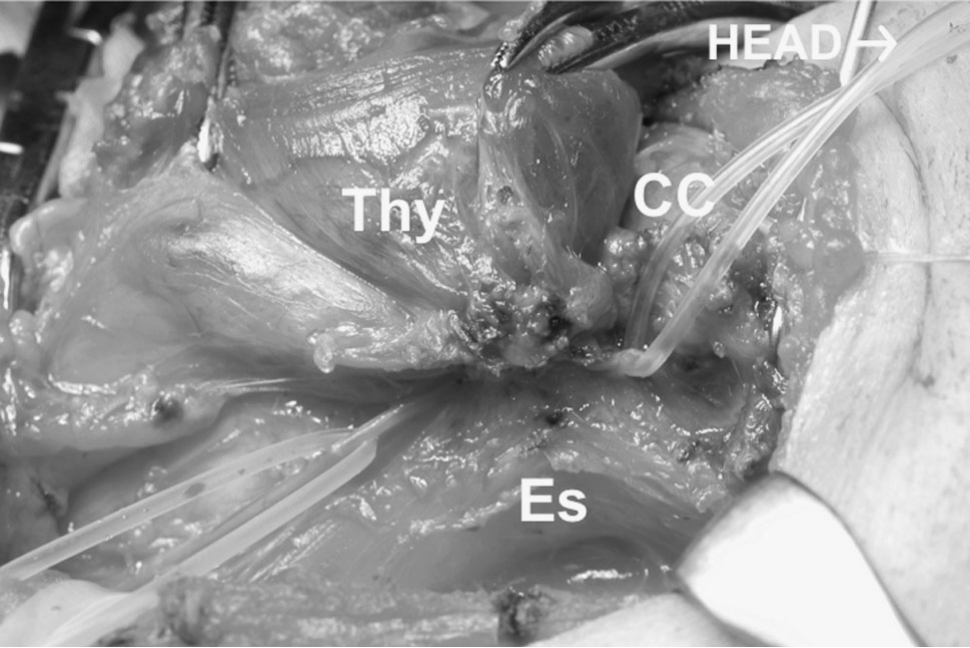
Thyroid cancer invading the RLN at Berry’s ligament. Silicon rubber tubes are holding the central portion of the nerve (*the left*) and the distal portion of the nerve (*the right*) that was found through the laryngeal approach dividing the inferior pharyngeal muscle. *Thy* the thyroid, *Es* esophagus. Head to the *right*

**Fig. 4:**
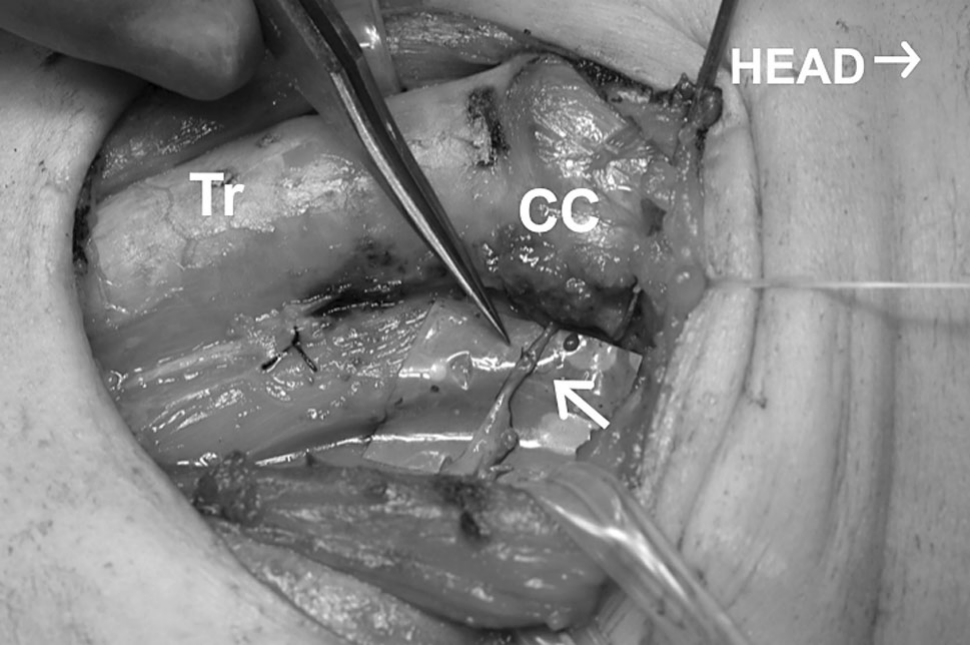
An ansa cervicalis-to-recurrent laryngeal nerve anastomosis was made following total thyroidectomy with central node dissection. Arrow indicates the anastomosis. The same patient shown in Fig. 3. Head to the *right*

Thyroid cancer often invades the RLN near Berry’s ligament. In such cases we used to divide the inferior pharyngeal constrictor muscle along the lateral edge of the thyroid cartilage to find the distal stump of the RLN, where we created an ansa-RLN anastomosis [4–6]. Finding the distal stump of the RLN was not easy, and we thus later modified our surgical procedure to perform this ***laryngeal approach*** before resecting the RLN with the cancer (Fig. 3) and to make an ansa-RLN anastomosis after resecting the tumor (Fig. 4); [10]. The distal stump behind the thyroid cartilage is thinner than the RLN outside its laryngeal entry point, making the anastomosis more difficult technically. In the present study, we classified the reconstruction cases into two groups according to the distal site of the anastomosis: outside or inside of the thyroid cartilage (349 and 145 cases, respectively) (Table 2).

### Evaluation of phonatory function

We performed laryngoscopic examinations periodically after surgery as well as preoperatively. However, laryngoscopic evaluation was not suitable for evaluating the recovery of phonatory function, since normal motion was not restored to the innervated vocal cords. We measured the maximum phonation time (MPT) periodically after surgery as described [7]. We also measured the mean airflow rate (MFR) during phonation to evaluate the patients’ vocal function, using a phonation analyzer (PA-1000, Minato Medical Science Co., Osaka, Japan) beginning in 2009. The MFR of the exhaled air during the phonation of the sound “a” at the volume of daily conversation was measured by the analyzer with the patient in a sitting position. MPT and MFR values at 1 year after surgery were used to evaluate the phonatory function outcomes.

A total of 1257 patients (1045 females and 212 males) with small thyroid cancers or thyroid nodules and normal laryngoscopic findings who were scheduled to undergo thyroid surgery served as normal controls in the present study, and 40 patients (29 females and 11 males) with unilateral VCP whose unilateral RLN had been resected due to thyroid cancer invasion but not reconstructed served as disease controls. The latter group included patients who underwent thyroid surgery at other hospitals. Patients with severe cancer invasion to the laryngopharyngeal region or with anaplastic thyroid cancer were excluded from the group of disease controls.

We analyzed the recovery of phonatory function following the RLN reconstruction in detail in relation to various factors, including the experience of the surgeons. In this study, we regarded 11 surgeons who had performed 10 or more RLN reconstructions as ‘experienced’ and 10 surgeons who had performed less than 10 reconstructions as ‘less-experienced.’ For these detailed analyses, we used female subjects only, since the numbers of male subjects were limited, and also to avoid gender differences in phonatory function. We chose the MPT for these detailed analyses, since we found that the MPT seemed more suitable than MFR as explained below in Results section. Statistical analyses were performed using the *t* test and Chi-square test. A *p*-value < 0.05 was regarded as significant.

## Results

**Fig. 5:**
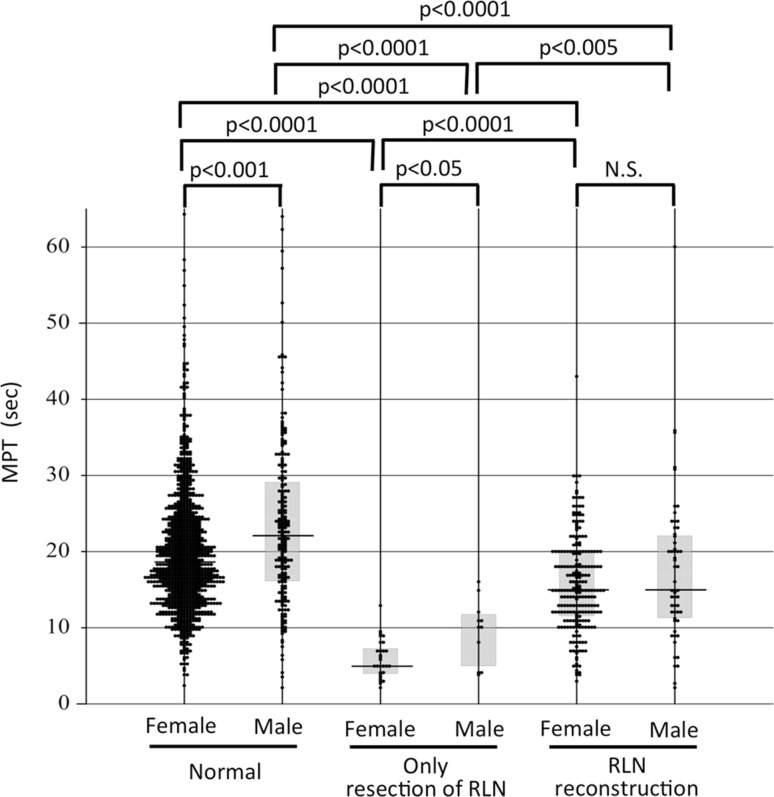
The MPTs of normal subjects, patients who had resection of the RLN without reconstruction, and patients who underwent reconstruction of the RLN. For the patients with RLN reconstruction, the MPT values 1 year after surgery are shown

Among the normal subjects, the men showed significantly longer MPTs than the women (median 22.2 and 18.6 s, respectively, *p* < 0.0001) (Fig. 5). The patients with VCP had significantly shorter MPTs than the normal subjects, in both genders (median 5.0 s in females and 10.0 s in males, *p* < 0.0001 and 0.0001, respectively). There was also a significant gender difference in MPT among the patients with VCP (*p* < 0.05). The patients who had undergone reconstruction of the RLN achieved significantly and substantially longer MPTs at 1 year after surgery (median values for females and males: 15.0 and 15.0 s, respectively) than the VCP patients, in both genders (*p* < 0.005). The MPTs at 1 year after surgery among the patients who underwent RLN reconstruction were close to the MPTs of the normal subjects, although there were small but significant differences in MPTs between these groups (*p* < 0.0001) (Fig. 5).

**Fig. 6:**
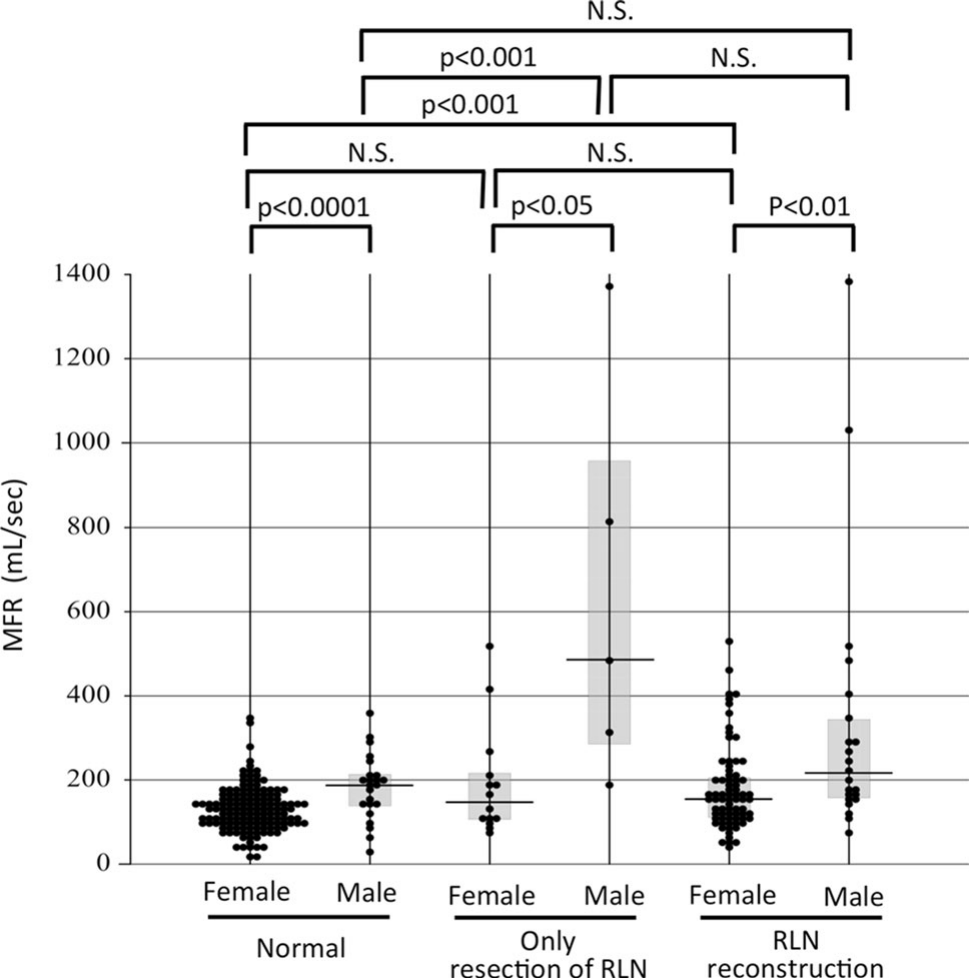
MFR values during phonation in normal subjects, patients who had resection of the RLN without reconstruction, and patients who underwent reconstruction of the RLN. For the patients with RLN reconstruction, the MFR values 1 year after surgery are shown

A smaller MFR may indicate better laryngeal ability to convert exhaled air into a voice. The male patients with VCP had significantly larger MFR values than the normal male subjects (median 486 and 189.0 mL/sec, respectively, *p* < 0.001) (Fig. 6). However, the difference in MFRs between the female VCP patients and the normal female subjects did not reach significance (median 149 and 127.0 mL/sec, respectively). In both genders, the differences in MFRs between the patients with VCP and the patients with RLN reconstruction did not reach significance (median MFRs in patients with RLN reconstruction: females: 157.0 mL/sec, males: 215.5 mL/sec, respectively). We had speculated that the MFR might provide an objective parameter of vocal cord function, but our findings indicated that the MFR was not superior to the MPT for evaluating phonatory function in these patient groups. This may be due to the small numbers of subjects who underwent evaluations with the phonation analyzer.

**Fig. 7:**
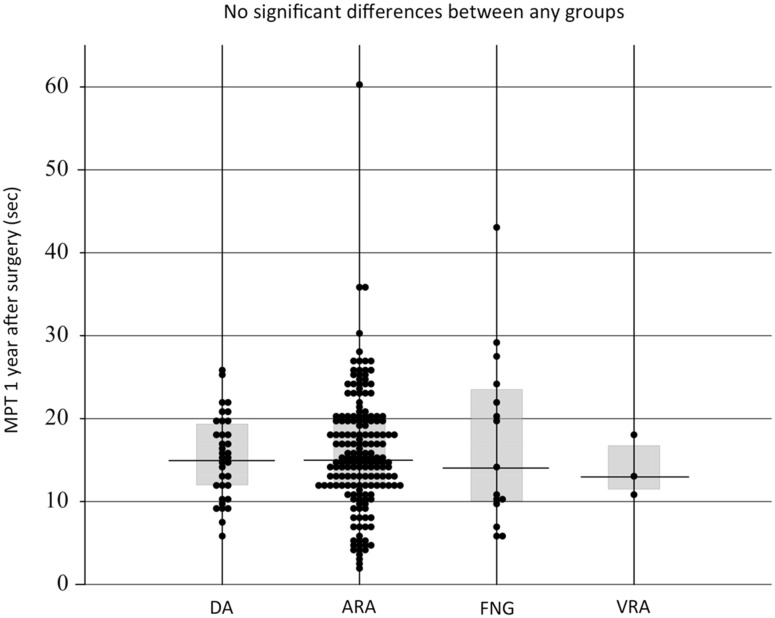
MPT values at 1 year after surgery in the female patients according to the methods of the reconstruction of the RLN. *DA* direct anastomosis, *ARA* ansa cervicalis-RLN anastomosis, *FNG* free nerve grafting, *VRA* vagus-RLN anastomosis

**Fig. 8:**
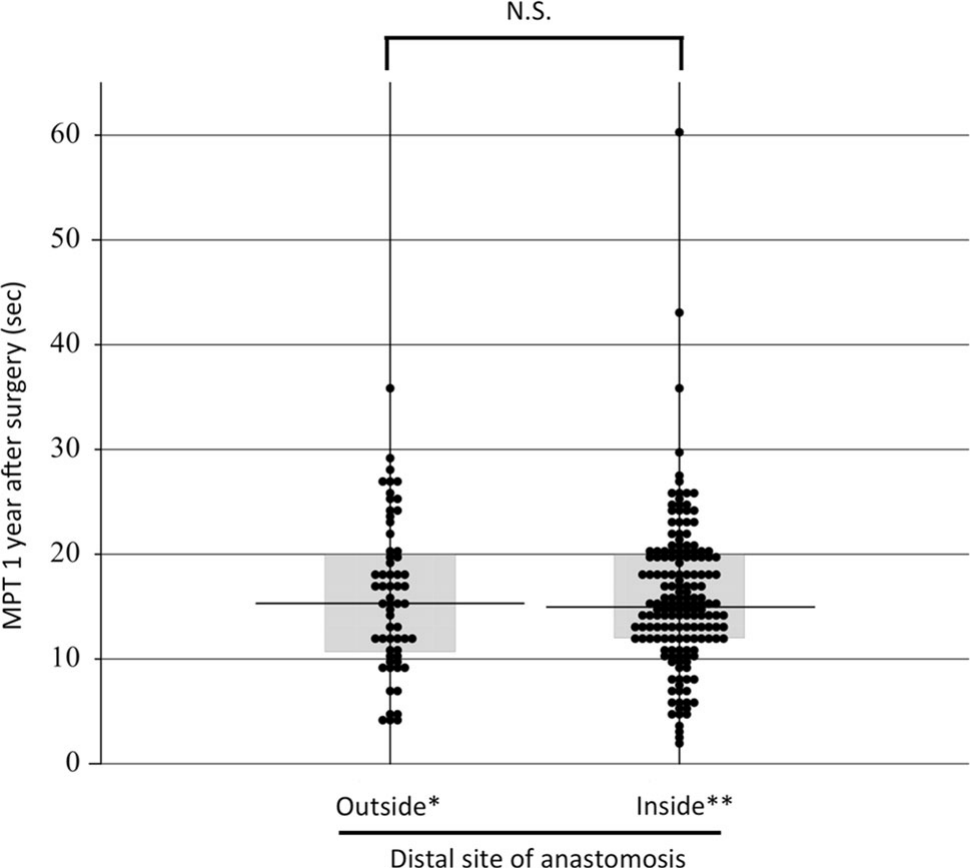
MPT values at 1 year after surgery according to the distal site of the anastomosis. Outside: the distal anastomosis was made outside of the thyroid cartilage; Inside: the distal anastomosis was made inside of the thyroid cartilage. The data are from female patients only

The main purpose of our study was to analyze in detail the factors that may affect the recovery of phonatory function following RLN reconstruction. We analyzed only the female subjects since there were clear gender differences in MPT, and because the number of male patients was small. In the group of female patients who underwent RLN reconstruction, there were no significant differences in MPT values at 1 year after surgery among the DA, ARA, FNG, and VRA subgroups (median 12.0, 12.0, 10.8, and 14.0 s, respectively) (Fig. 7). There was also no significant difference according to the distal site of anastomosis, either inside or outside the thyroid cartilage (median 15.4 and 15.0 s, respectively) (Fig. 8), although the former anastomosis technique was technically more difficult to perform than the latter.

**Fig. 9:**
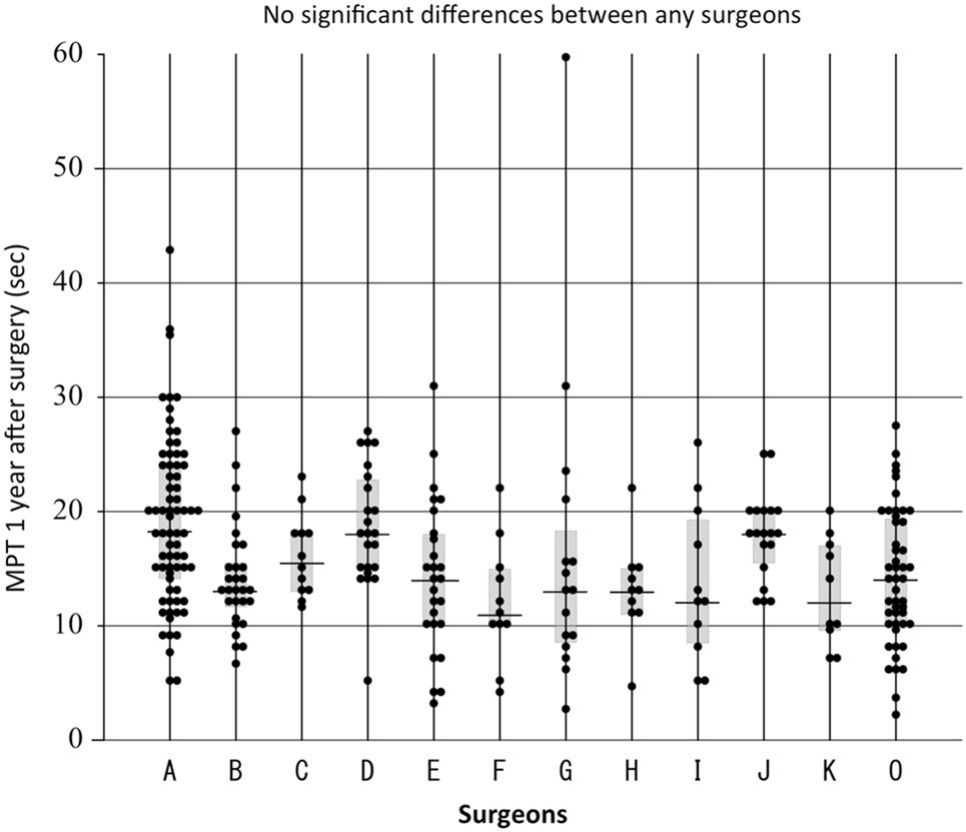
MPT values at 1 year after surgery according to the surgeon who performed the reconstruction. ‘A’ to ‘K’ indicate the 11 experienced surgeons who had performed 10 or more RLN reconstructions, and ‘O’ indicates the group of 10 less-experienced surgeons who had performed less than 10 reconstructions

There were also no significant differences in the phonatory outcomes of the patients according to the surgeons who performed the reconstruction in our comparison of the group of 11 experienced surgeons and the group of 10 less-experienced surgeons (Fig. 9). Age, the preoperative presence or absence of VCP, the use of operative magnification, the thickness of the thread used for the anastomosis, and the number of sutures used to make the anastomosis also did not significantly affect the MPT at 1 year after surgery (Table 3).

**Table 3:** Maximum phonation time according to various factors in female patients only

Factor	No. of patients *n* (%)	MPT (sec)	Significance
Age			
<60 years	104 (46.4)	14.0	N.S.
≥60 years	124 (53.6)	16.0	
VCP preoperatively			
Yes	104 (46.4)	11.0	N.S.
No	124 (53.6)	12.0	
Use of operative magnification			
Surgical loupes	124 (82.1)	15.5	N.S.
None	27 (17.9)	17.0	
Suture thread thickness			
7–0	3 (1.4)	17.3^*^	
8–0	206 (96.7)	15.0	
9–0	4 (1.9)	13.0^*^	
Number of sutures			
1	8	9.0	N.S.
2	78	12.0	
3	64	11.5	
4	3	16.6^*^	

Median values are shown except for cases with ^*^ for which mean values are given

*MPT* maximum phonation time, *VCP* vocal cord paralysis

An MPT value <9 s in Japanese adult females is regarded as insufficient vocal cord function that could cause some inconvenience in daily verbal communication [11]. Among the present 228 female patients for whom MPT data at 1 year after surgery were available, 28 patients (12.3 %) had MPTs <9 s. The ratio of the patients with insufficient recovery following RLN reconstruction was not associated with the method of RLN reconstruction, the preoperative presence or absence of VCP, or the site of the distal anastomosis (Table 4); nor was it associated with age, the use of a magnifier, the thickness of the suture thread, or the surgeon who performed the surgery (data not shown).

**Table 4:** Factor analysis of the insufficient recovery in phonation in the female patients who underwent RLN reconstruction

Factor	MPT ≥9 s *n* (%)	MPT <9 s *n* (%)	Significance
Method of RLN reconstruction			
Direct anastomosis	33 (94.3)	2 (5.7)	N.S.
Ansa cervicalis-RLN anastomosis	152 (86.9)	23 (13.1)	
Free nerve grafting	12 (80.0)	3 (20.0)	
VCP preoperatively			
Yes	92 (88.5)	12 (11.5)	N.S.
No	108 (87.5)	16 (12.9)	
Site of distal anastomosis			
Outside	146 (87.4)	**21 (12.6)**	N.S.
Inside	54 (88.5)	7 (11.5)	

Outside: outside of the thyroid cartilage; Inside: inside the thyroid cartilage

## Discussion

We have reported that the voices of the patients who underwent RLN reconstruction recovered following the surgery, although the vocal cords on the side of the reconstruction remained immobile [4–7]. Our methods of RLN reconstruction included DA, ARA, FNG, and VRA. In a previous study, we observed that the phonatory recovery following RLN reconstruction occurred irrespective of gender, the age of the patient, the preoperative presence or absence of VCP, the method of reconstruction, the use of a magnifier for the reconstruction, and the thickness of the suture thread [7]. Those reported cases, however, were operated on by a single surgeon (A. Miyauchi). In the present report, we included all 449 cases of RLN reconstruction performed by 21 surgeons at Kuma Hospital over a 17-year period.

The results of the present analyses confirmed that phonatory recovery occurred following the RLN reconstructions. The patients who underwent RLN reconstruction achieved nearly normal values of MPT as evaluated at 1 year after surgery. However, these values were slightly but significantly lower than those of the normal subjects when analyzed in the large number of patients in the present series.

In this study, we analyzed the possible effects of various factors on MPT 1 year after surgery in female patients who underwent RLN reconstruction. The results confirmed those of our previous report, which showed that age, the preoperative presence or absence of VCP, the method of reconstruction, the use of surgical magnifiers, and the thickness of the suture thread did not significantly affect MPT 1 year after surgery. In addition to these findings, we were able to show that phonatory recovery following anastomosis inside the thyroid cartilage was not inferior to that following anastomosis outside the thyroid cartilage, although the former procedure is technically more difficult to perform than the latter, since the nerve stumps there are much thinner than the RLNs outside the thyroid cartilage.

We observed that phonatory recovery was also not dependent on the experience of the surgeons who performed the reconstruction. This seems to be different than in the case of vascular anastomosis, in which poor techniques can result in poor outcomes, leakage, or obstruction [12]. The axons of the proximal nerve regenerate from the proximal nerve end to the distal nerve stump following the anastomosis. When both ends are close to each other, the regenerating axons grow into the distal nerve stump by themselves. Less than 100 % reinnervation might be sufficient to obtain a recovery sufficient for daily phonation. Of course, we should do our best to make a precise end-to-end anastomosis during surgery.

Although the majority of our patients who underwent RLN reconstruction achieved phonatory recovery sufficient for daily verbal communication, 12.3 % of the female patients had an MPT of <9 s at 1 year after surgery, indicating insufficient recovery. This insufficient recovery was not associated with the factors mentioned above. One possible cause for this might be the separation of the anastomosis of the nerve ends. This could have occurred in some cases. However, our present findings clarified that the insufficient recovery was not associated with surgical issues such as the method of reconstruction, surgical techniques, or the experience of the surgeon, indicating that separation of the anastomosis was likely not the main reason. Another possibility might be an extreme extent of misdirected reinnervation or uneven reinnervation for the adductor nerves and abductor nerves.

Before analyzing the present data, we speculated that the phonatory recovery following anastomosis inside the thyroid cartilage might be worse than that following anastomosis outside the thyroid cartilage, because the former procedure is technically more difficult. However, our findings revealed that the results of the anastomoses inside the thyroid cartilage were unexpectedly similar to those of the anastomoses outside the thyroid cartilage. The RLN branches to the adductor muscles and the abductor muscles behind the thyroid cartilage. The anastomosis inside the thyroid cartilage might have been done selectively with the branch to the adductor muscles. This might have resulted in a preferable recovery in phonation. Further studies with electromyographic examinations are necessary to determine whether this was the case.

In conclusion, improvement in phonation was obtained in approximately 90 % of the patients who needed resection of the RLN regardless of age, gender, or vocal cord status when the nerve reconstruction was performed with any type of reconstruction modality using any surgical technique. Performing the reconstruction during thyroid surgery is essential in cases of RLN resection. All thyroid surgeons should be familiar with these techniques.
